# Anchoring the snare tip is a feasible endoscopic mucosal resection method for small rectal neuroendocrine tumors

**DOI:** 10.1038/s41598-021-92462-y

**Published:** 2021-06-21

**Authors:** Jeongseok Kim, Jisup Kim, Eun Hye Oh, Nam Seok Ham, Sung Wook Hwang, Sang Hyoung Park, Byong Duk Ye, Jeong-Sik Byeon, Seung-Jae Myung, Suk-Kyun Yang, Seung-Mo Hong, Dong-Hoon Yang

**Affiliations:** 1 Department of Gastroenterology, Asan Medical Center, University of Ulsan College of Medicine, 88, Olympic-ro 43-gil, Songpa-gu, Seoul, 05505 Korea; 2 Department of Internal Medicine, Keimyung University School of Medicine, Daegu, Korea; 3 Department of Pathology, Asan Medical Center, University of Ulsan College of Medicine, Seoul, Korea; 4 Department of Gastroenterology, Haeundae Paik Hospital, Inje University College of Medicine, Busan, Korea

**Keywords:** Colonoscopy, Gastrointestinal diseases

## Abstract

Small rectal neuroendocrine tumors (NETs) can be treated using cap-assisted endoscopic mucosal resection (EMR-C), which requires additional effort to apply a dedicated cap and snare. We aimed to evaluate the feasibility of a simpler modified endoscopic mucosal resection (EMR) technique, so-called anchored snare-tip EMR (ASEMR), for the treatment of small rectal NETs, comparing it with EMR-C. We retrospectively evaluated 45 ASEMR and 41 EMR-C procedures attempted on small suspected or established rectal NETs between July 2015 and May 2020. The mean (SD) lesion size was 5.4 (2.2) mm and 5.2 (1.7) mm in the ASEMR and EMR-C groups, respectively (p = 0.558). The en bloc resection rates of suspected or established rectal NETs were 95.6% (43/45) and 100%, respectively (p = 0.271). The rates of histologic complete resection of rectal NETs were 94.1% (32/34) and 88.2% (30/34), respectively (p = 0.673). The mean procedure time was significantly shorter in the ASEMR group than in the EMR-C group (3.12 [1.97] vs. 4.13 [1.59] min, p = 0.024). Delayed bleeding occurred in 6.7% (3/45) and 2.4% (1/41) of patients, respectively (p = 0.618). In conclusion, ASEMR was less time-consuming than EMR-C, and showed similar efficacy and safety profiles. ASEMR is a feasible treatment option for small rectal NETs.

## Introduction

Rectal neuroendocrine tumor (NET) is the second most common gastrointestinal NET after small bowel NET, and accounts for 34% of all gastrointestinal NETs^1^. The incidence and prevalence of rectal NET have increased over time because of the widespread use of colonoscopy for colorectal cancer screening^2,3^. Rectal NET has an excellent prognosis, with a median overall survival of 24.6 years, probably due to it being discovered as a small low-grade lesion localized to the submucosa in the majority of cases; however, it can be incurable once it has progressed to unresectable metastatic disease^2,4,5^.

Early-stage small rectal NET without evidence of lymph node or distant metastasis can be successfully treated by endoscopic resection^6–8^. Endoscopic submucosal dissection (ESD) is an effective resection technique for small rectal NETs, as it can achieve a higher rate of histologic complete resection than conventional endoscopic mucosal resection (EMR)^9–11^. However, the ESD procedure requires a high degree of expertise and carries a higher risk of adverse events (such as perforation and bleeding) than conventional EMR^12^. In comparison with ESD, modified EMR techniques such as cap-assisted EMR (EMR-C), EMR with band ligation (EMR-L), and EMR with circumferential precutting (EMR-P) are relatively simple^13,14^, and can thus be performed by therapeutic endoscopists who are not proficient at ESD. Our previous study on small rectal NETs indicated that EMR-C required a shorter procedure time than ESD, while the therapeutic efficacy and safety profiles did not significantly differ between the two procedures^13^. Furthermore, a recent meta-analysis suggested that EMR with suction (EMR-C or EMR-L) was superior to ESD for complete removal of small rectal NETs^15^. However, EMR with suction techniques requires specialized devices, such as a band ligation device for EMR-L and a suction cap with inner rim looped by a crescent-type snare for EMR-C. As these devices are essential for each procedure, endoscopists and assistants need to spend additional time and effort on applying the devices to the scope.

Anchored snare-tip EMR (ASEMR), or tip-in EMR is a technique that anchors the snare tip into a small mucosal slit to prevent the snare from being slipped while capturing a lesion^16,17^. Previous studies showed that ASEMR increased specimen size and facilitated complete resection of flat or large colorectal neoplasia^18–20^. Deep resection of rectal NETs may be possible with the ASEMR technique, given that the mucosal slit (anchor point) and pressing power on the snare toward the anchor point can work as the “fulcrum” of a lever and the “effort motion” on it, respectively. However, there is no published study on the use of ASEMR for rectal NETs. Here, we compared the procedural outcomes of ASEMR with those of EMR-C for the treatment of small suspected or established rectal NETs to evaluate the feasibility of ASEMR.

## Methods

### Patient selection

**Figure 1:**
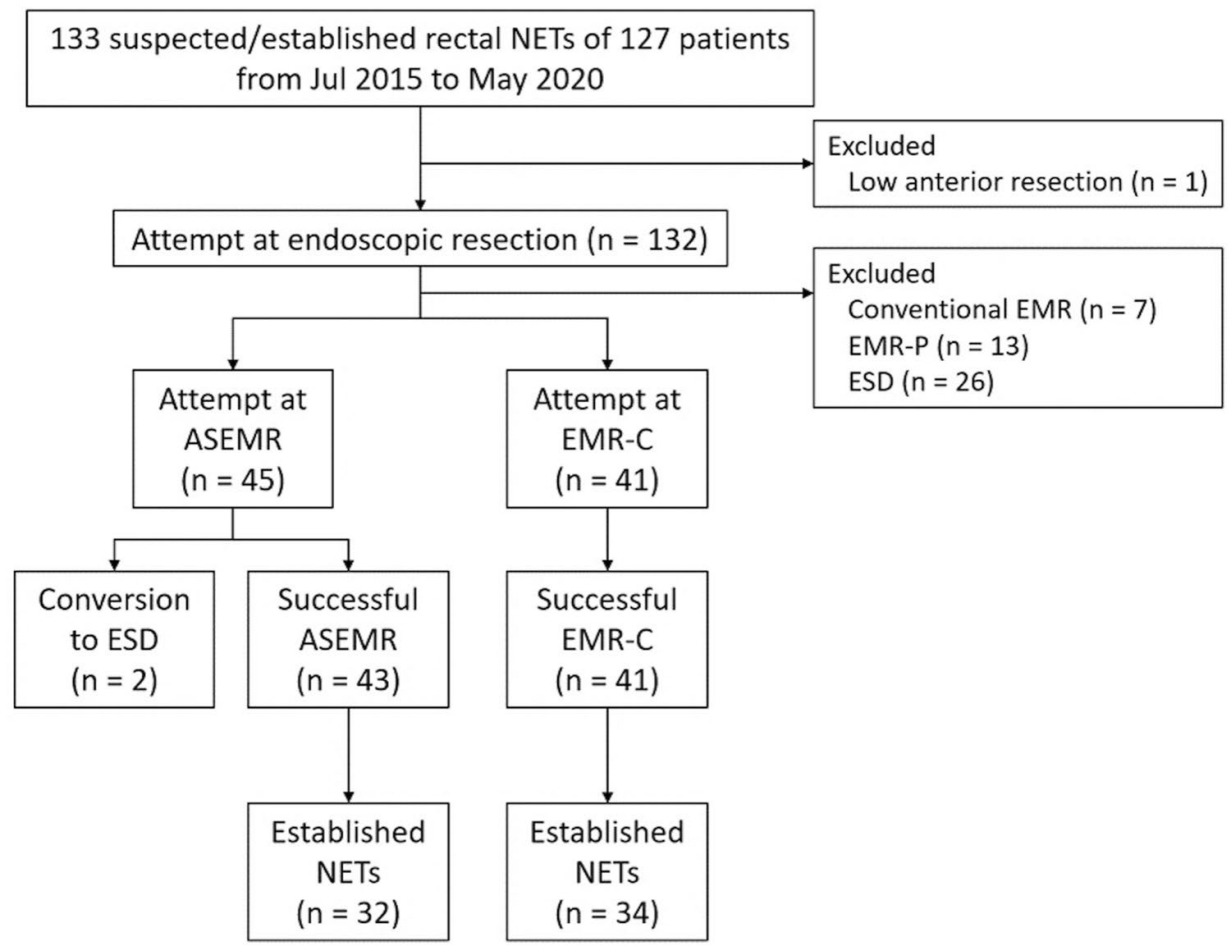
Flow chart of patient throughput. *NET* neuroendocrine tumor, *EMR* endoscopic mucosal resection, *EMR-P* EMR with circumferential precutting, *ESD* endoscopic submucosal dissection, *ASEMR* anchored snare-tip EMR, *EMR-C* cap-assisted EMR.

By conducting retrospective chart review, we recognized a total of 127 patients having 133 suspected or established rectal NETs were referred for endoscopic treatment from July 2015 to May 2020. Abdominopelvic computed tomography was performed for newly diagnosed rectal NET cases. Anorectal endoscopic ultrasonography (EUS) was selectively performed to determine whether the NET was confined to the submucosal layer or not in patients with NET > 10 mm in size or NET with depressed or ulcerated surface. A patient who had a rectal NET with suspected proper muscle invasion on EUS underwent a low anterior resection. Endoscopic resection was performed for the remaining 132 lesions in 126 patients by a single endoscopist (D.H.Y.) who had expertise in EMR, EMR-P, EMR-C, and ESD^13,14^. EMR-C was mainly conducted before March 2019, and ASEMR was conducted thereafter because of the reimbursement issue. ASEMR was attempted for 45 lesions, and EMR-C was performed on 41 lesions (Fig. 1). As conventional EMR (n = 7) and EMR-P (n = 13) were performed in only a limited number of cases, ASEMR was not compared with these resection methods. As ESD (n = 26) was performed for NETs larger than 10 mm or those having a surface depression or ulceration, ASEMR and ESD were also not compared.

### Ethical approval

The study protocol followed the ethical guidelines of the Declaration of Helsinki Principles, and was approved by the institutional review board of Asan Medical Center (IRB number 2019–1510). All patients provided informed consent for the endoscopic resection procedure, and the institutional review board of Asan Medical Center approved the request to waive the documentation of informed consent for this retrospective study.

### Equipment and sedation for endoscopic resection

A high definition colonoscopy system (CF-HQ290AI, EVIS LUCERA III, Olympus Medical, Tokyo, Japan; EC-3890Zi, Optivista EPKi-7070, Pentax Medical, Tokyo Japan; or EC-760R-VM, ELUXEO-7000, Fujifilm, Tokyo, Japan) was used for ASEMR and EMR-C. An electrosurgical unit (VIO 300D, Erbe Elektromedizin GmbH, Tuebingen, Germany) was used for both procedures. Both ASEMR and EMR-C were performed under mild to moderate sedation using intravenous midazolam.

### ASEMR technique

**Figure 2:**
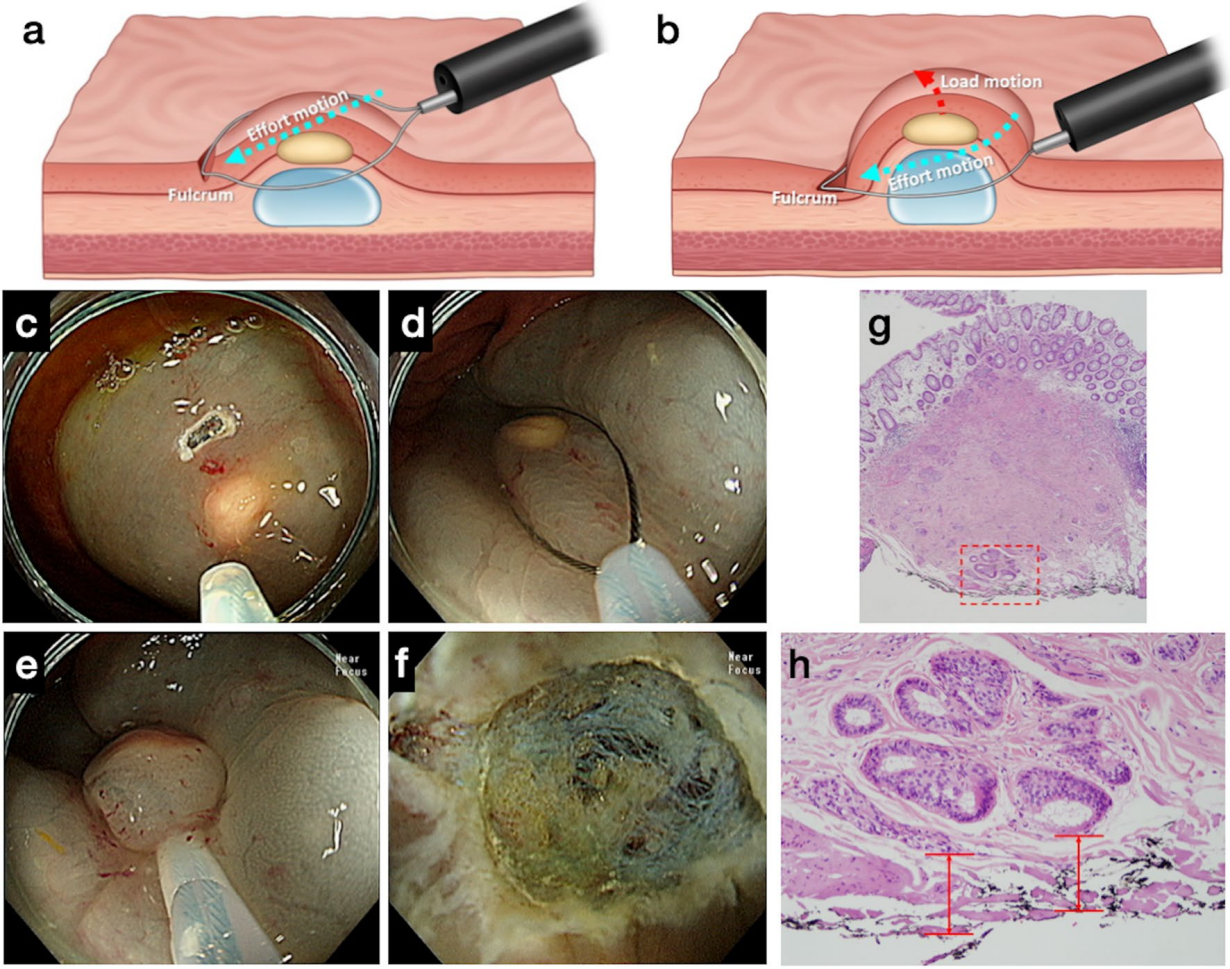
Principles of anchored snare-tip endoscopic mucosal resection for a suspected or established rectal neuroendocrine tumor (NET). (**a**) The snare-tip is anchored into the mucosal incision site on the oral side of the lesion. The anchoring point works as a fulcrum for leverage while the snare-tip is gently pressed toward the direction indicated by the cyan dotted arrow (effort motion). (**b**) The NET will rise slightly toward the luminal side (red dotted arrow) because of a load motion resulting from the leverage effect. The anchoring should be maintained by pressing the snare-tip toward the mucosal incision site while tightening the snare (continued effort motion toward the direction indicated by the cyan dotted arrow). During snaring, the snare sheath can be pressed downward to ensure en bloc snaring (see the curved tail part of the cyan dotted arrow). (**c**) A mucosal incision was made on the oral side of the lesion. (**d**) The snare was anchored while being opened. (**e**) Anchoring was well maintained until en bloc snaring was completed. (**f**) En bloc complete resection was possible. (**g**), (**h**) The deep safety resection margin was measured after endoscopic resection.

**Figure 3:**
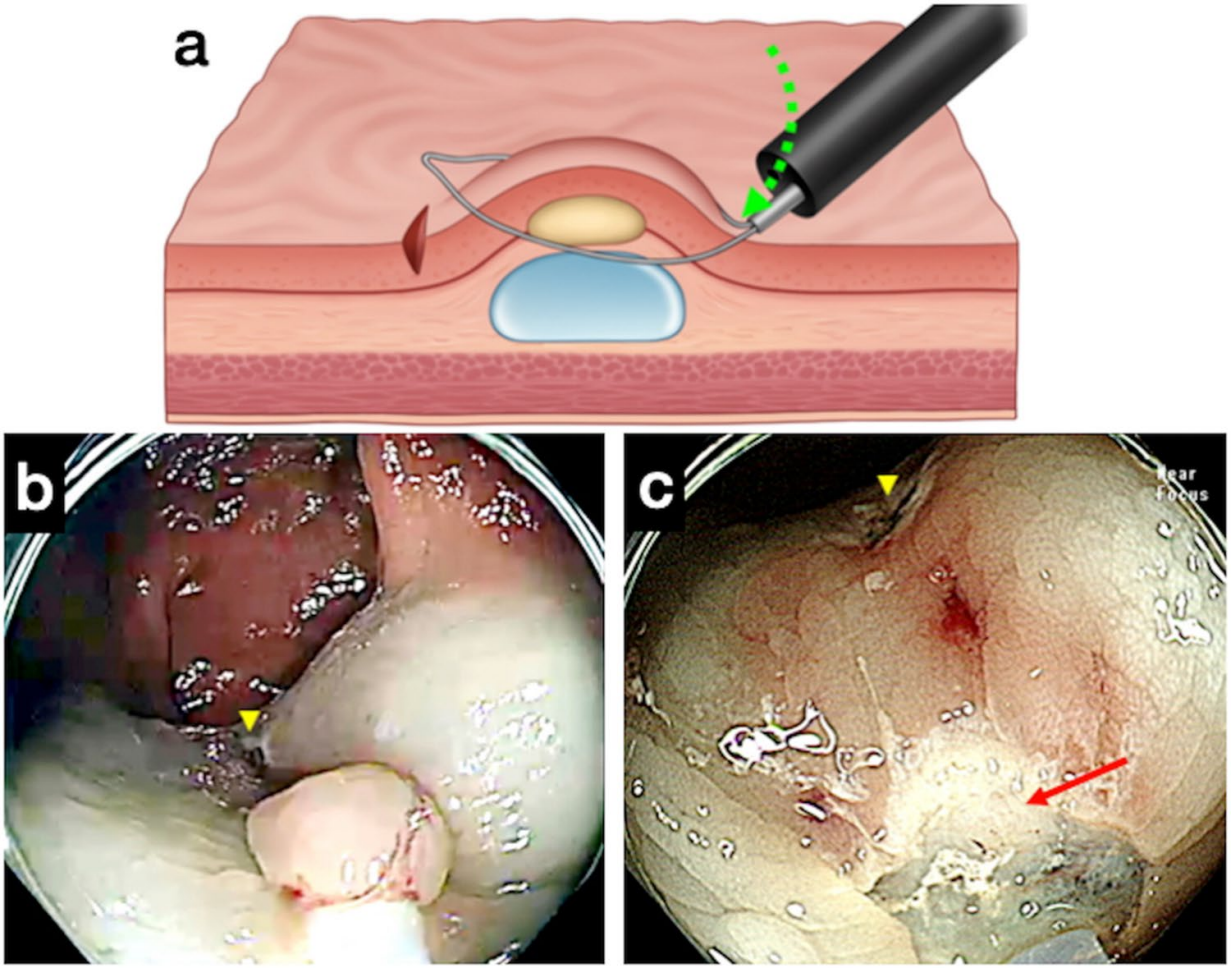
A failed case of anchored snare-tip endoscopic resection for an established rectal neuroendocrine tumor (NET). (**a**) If the anchoring is loosened because of insufficient pressure toward the anchoring point (see the green dotted arrow), the snare will slip at the oral side of the lesion and the rise of the neuroendocrine tumor (NET) secondary to the load motion will not happen. (**b**) Anchoring was not maintained during snaring, and the snare slipped on the oral side. The yellow arrowhead indicates the mucosal incision site for anchoring. (**c**) Remnant NET tissue was observed after the anchored snare-tip endoscopic mucosal resection (see the red arrow).

A conventional transparent cap was attached to the distal end of the colonoscope. A 13 mm oval type stiff snare with a 0.42 mm wire diameter (Captivator, Boston Scientific, Marlborough, MA, USA) was used in 33 ASEMR attempts, and a 15 mm hexagonal snare with a 0.25 mm wire diameter (FSH, Endo-Upex, Upex-med, Anyang, Korea) was used in one ASEMR attempt. Indigo carmine-tinted saline and epinephrine solution (1:100 000) was injected submucosally around the lesion. A small mucosal slit was made 3–5 mm proximal to the lesion using the tip of the snare under Endocut Q mode (effect 3, duration 2, interval 6). Next, the snare tip was anchored into the mucosal slit, and the snare was slowly opened and the lesion gently entrapped. While the snare was being tightened, it was slightly pressed toward the anchoring point to maintain its width. Lesions located in the submucosal layer could be lifted superficially by pressing toward the anchoring point because the mucosal slit (anchoring point) works as the fulcrum of a lever, and the pressing power acts as the effort motion on the lever (Fig. 2, Supplementary Video 1). Lesions were cut using the Endocut Q mode after en bloc snaring. For ASEMR, the angle of the snare-sheath axis is important, as the effort motion on the lever will be transmitted through the snare-sheath axis to the anchoring point (fulcrum). This angle can be either horizontal or more angulated while ASEMR is being performed (Fig. 3).

### EMR-C technique

After submucosal injection using the same solution as used for ASEMR, a crescent-type snare (SD-221L-25; Olympus Medical) was looped along the inner rim of an EMR-C-dedicated cap (MH-597; Olympus Medical). The lesion was then suctioned into the cap, grasped using the snare, and resected. The resection with EMR-C used the same Endocut Q mode used for ASEMR.

### Data collection and outcome measures

Demographic variables including age, sex, presence of coagulopathy, and history of antiplatelet or anticoagulant medication (warfarin and direct oral anticoagulants) were retrospectively retrieved from the electronic medical records. Laboratory data such as platelet count, prothrombin time, and activated partial thromboplastin time measured within 6 months before the procedures were also collected.

The endoscopy results and pathology reports were retrospectively reviewed. Technical success of the procedure was defined as gross en bloc resection by the primarily selected resection technique. In cases of technical failure, the mechanism of failure was prospectively recorded, and the originally selected resection technique was immediately converted to ESD as a rescue procedure. Resection time was defined as the time from the initiation of submucosal injection to gross complete removal of the lesion. The pathology of the resected specimens was reviewed by two board-certified pathologists (S.M.H., J.K.). The status of lateral and deep resection margins was evaluated for the neoplastic lesions, which were mainly NETs. The deep safety resection margin, defined as the shortest distance from the lower border of the NET to the distal end of the specimen, was measured. Delayed bleeding was defined as any bleeding from the resection site that required endoscopic hemostasis or transfusion after completion of endoscopic procedures. Although measures prevent delayed bleeding were not routinely performed, argon plasma coagulation or clipping is selectively performed to control immediate postpolypectomy bleeding. Perforation was defined as an endoscopically identified mural defect or perirectal fat exposure in the rectum. The horizontal faced position weight was measured at a 45° angle to assess the stiffness of the two snares used in this study^21^.

### Statistical analysis

Chi-square or Fisher’s exact tests were performed for comparative analysis of categorical variables. Continuous variables were analyzed using Student’s *t*-test. The correlation between the pathology-determined size of the NETs and the deep safety resection margins was analyzed using Spearman’s correlation coefficient. A p value < 0.05 was considered statistically significant. SPSS version 21.0 for Windows software (SPSS Inc., Chicago, IL, USA) was used for statistical analysis.

## Results

### Comparison between ASEMR and EMR-C for small suspected or established rectal NETs

The mean age of the ASEMR group was older than that of the EMR-C group (50.9 [10.2] vs. 43.6 [11.7] years, p = 0.004). All EMR-C procedures were successfully completed, but two of the 45 attempted ASEMR procedures failed to achieve gross en bloc resection owing to slippage of snares. These two failed ASEMR cases were immediately converted to ESD. Therefore, the technical success rates were 95.6% for ASEMR and 100% for EMR-C (p = 0.271). The mean resection time of the successful procedures was significantly shorter in ASEMR than in EMR-C (2.80 [0.87] vs. 4.57 [2.31] min, p < 0.001). The incidence of delayed bleeding was not significantly different between the two groups (6.7% vs. 2.4%, p = 0.618). The patient and lesion characteristics, and procedural outcomes are described in detail in Table 1.

**Table 1:** Comparison of ASEMR (n = 45) versus EMR-C (n = 41) for suspected or established rectal NETs.

	ASEMR (n = 45)	EMR-C (n = 41)	p value
No. of lesions / patients	45 / 41	41 / 38	
Patient characteristics			
Age in years, mean (SD)	50.9 (10.2)	43.6 (11.7)	0.004
Male, n (%)	24 (58.5%)	21 (55.3%)	0.769
Platelet count, × 10^3^/µL, mean (SD)	254.9 (63.6)	265.1 (54.6)	0.450
PT, INR, mean (SD)	0.99 (0.05)	1.01 (0.06)	0.074
aPTT, s, mean (SD)	27.4 (2.1)	27.6 (2.4)	0.691
Antiplatelet medication, n (%)	1 (2.2%)	0	1.000
Warfarin or DOAC, n (%)	0	0	NA
Lesion characteristics			
Failed endoscopic resection by referring endoscopists, n (%)	3 (6.7%)	7 (17.1%)	0.183
Preprocedural diagnosis, n (%)			0.280
Suspected NET	25 (55.6%)	18 (43.9%)	
Established NET	20 (44.4%)	23 (56.1%)	
Endoscopic size, mm, mean (SD), [range]	5.4 (2.2) [2–13]	5.2 (1.7) [3–10]	0.558
Procedure outcomes			
Technical success, n (%)	43 (95.6%)	41 (100%)	0.271
Resection time for successful cases, min, mean (SD)	2.80 (0.87)^a^	4.57 (2.31)	< 0.001
Resection time for overall cases, min, mean (SD)	3.10 (1.83)	4.57 (2.31)	0.002
Final histology, n (%)			0.401
NET	34 (75.6%)	34 (82.9%)	
No remnant NET or not NET	11 (24.4%)	7 (17.1%)	
Delayed bleeding, n (%)	3 (6.7%)^b^	1 (2.4%)^c^	0.618
Perforation, n (%)	0	0	NA

*ASEMR* anchored snare-tip endoscopic mucosal resection, *EMR-C* cap-assisted endoscopic mucosal resection, *NET* neuroendocrine tumor, *SD* standard deviation, *PT* prothrombin time, *INR* international normalized ratio, *aPTT* activated partial thromboplastin time, *DOAC* direct oral anticoagulant, *NA* not applicable.

^a^Two failed ASEMR cases were excluded because of direct conversion to ESD during the procedures.

^b^Three patients experienced delayed bleeding in ASEMR group. One patient with 6 mm-sized rectal NET failed ASEMR attempt due to slippage of snare, and ASEMR was immediately converted to ESD. The other two patients showed scar change and lymphoid polyp in the final histology results, respectively.

^c^One patient with 3 mm-sized rectal NET experienced delayed bleeding.

### Details of the failed ASEMR procedures

A thin and less-stiff snare was applied for one ASEMR attempt, which failed because of slippage of the snare (Supplementary Video 2). The horizontal faced position weight at a 45° angle was measured as 2.96 g for the thin-wired snare and 16.1 g for the stiff snare. Less-stiff snares were not further used, as the stiffness was considered essential for capturing a lesion in an en bloc manner for ASEMR. A stiff snare was therefore applied in 44 ASEMR attempts, with one of these failing because of slippage of the snare. The video clip of this failed ASEMR case demonstrates slippage of the anchored snare-tip at the oral side of the lesion, slippage that was not noticed during the procedure (Fig. 3, Supplementary Video 2). Rescue ESD procedures for the two failed ASEMR cases were successfully completed in the same sessions.

### Procedural and histologic outcomes of rectal NETs resected by ASEMR and EMR-C

Thirty-four lesions were identified as NETs on the final histopathology in ASEMR group and EMR-C group, respectively. Among 18 patients (20.9%) who showed non-NETs in the final histology results, scar change was observed in 4 and 7 patients in ASEMR and EMR-C groups, respectively. Lymphoid polyps (n = 4), hypertrophy of the muscularis mucosa (n = 1), fat necrosis (n = 1), and a granular cell tumor (n = 1) were also observed in ASEMR group. Among established NETs, there was no significant difference in lesion size, histologic complete resection rate, and deep safety resection margin between the ASEMR and EMR-C groups (Table 2). The pathology-determined size of the NETs and deep safety resection margins were not correlated in either the ASEMR (Spearman r = 0.111, p = 0.532) or EMR-C (Spearman r =  − 0.038, p = 0.832) groups. Rectal NETs larger than 10 mm on pathology-determined size were observed in only two patients (11 and 12 mm), and they were successfully resected by ASEMR with deep safety resection margins of 230 and 1900 μm, respectively. The mean resection time of rectal NETs was significantly shorter in the ASEMR group than in the EMR-C group (3.12 [1.97] vs. 4.13 [1.59] min, p = 0.024, Table 2).

**Table 2:** Outcomes of established rectal NETs resected by ASEMR (n = 34) and EMR-C (n = 34).

	ASEMR (n = 34)	EMR-C (n = 34)	p value
Endoscopic size, mm, mean (SD), [range]	5.7 (2.3) [2–13]	5.5 (1.7) [3–10]	0.672
Pathologic size, mm, mean (SD) [range]	5.4 (2.5) [1–12]	4.9 (1.7) [2–10]	0.402
Grade, n (%)			0.356
Grade 1	33 (97.1%)	30 (88.2%)	
Grade 2	1 (2.9%)	4 (11.8%)	
Lymphovascular invasion, n (%)	0	0	NA
Resection time, min, mean (SD)	3.12 (1.97)	4.13 (1.59)	0.024
Histologic complete resection, n (%)	32 (94.1%)	30 (88.2%)	0.673
Clear lateral margin, n (%)	34 (100%)	32 (94.1%)	0.357
Clear deep margin, n (%)	32 (94.1%)	31 (91.2%)	0.365
Deep safety resection margin, µm, mean (SD)	291.8 (347.7)	259.7 (262.2)	0.669
Delayed bleeding, n (%)	1 (2.9%)	1 (2.9%)	1.000
Perforation, n (%)	0	0	NA

*ASEMR* anchored snare-tip endoscopic mucosal resection, *EMR-C* cap-assisted endoscopic mucosal resection, *NET* neuroendocrine tumor, *SD* standard deviation, *NA* not applicable.

## Discussion

According to international guidelines, referring to multidisciplinary tumor board in a dedicated center is recommended to determine the best treatment strategy for the patients with rectal NET^22,23^. In the current study, en bloc resection was possible in 95.6% of suspected and established rectal NETs, and histologic complete resection was achieved in 94.1% of rectal NETs using the ASEMR method. In our retrospective dataset, both the en bloc and complete resection rates of ASEMR were not significantly different from those of EMR-C. Compared with the complete resection rate of EMR-C for small NETs found in our previous report (94.1%)^13^, and the pooled complete resection rate of EMR with suction methods for small NETs reported in a meta-analysis (93.7%)^15^, the complete resection rate of ASEMR for rectal NETs found in the current study seems acceptable. According to an earlier report on the ASEMR technique, the provision of gentle pressure toward the anchoring point helps the snare widen laterally while snaring and contributes to acquisition of a larger specimen size^18^. For the complete removal of small rectal NETs involving the submucosal layer, the thickness of the resected specimen or deep safety resection margin are more important than the lateral size of the specimen. The deep safety resection margins of the specimens provided by the two resection methods showed no significant differences between the two groups (291.8 [347.7] µm in the ASEMR group vs. 259.7 [262.2] µm in the EMR-C group). The pressure toward the anchor point in the ASEMR method may help to resect small NETs as deeply as in the EMR-C method, probably by moving the NETs upward with the leverage action (Fig. 2). Deeper resection may expose patients to additional risk of delayed bleeding and perforation; however, in this study the incidence of bleeding was not significantly different between the two types of procedures, and no perforation occurred. Of the three patients with delayed bleeding in ASEMR group, one patient with 6 mm-sized rectal NET was failed an ASEMR attempt due to slippage of snare, and ASEMR was immediately converted to ESD. The other 2 patients showed scar change and lymphoid polyp in the final histology results, respectively. In EMR-C group, one patient with 3 mm-sized rectal NET experienced delayed bleeding. As all procedures were performed for lesions located in the rectum, the risk of perforation might have been minimal. Therefore, for the treatment of small suspected or established rectal NETs, we consider ASEMR and EMR-C to be equivalent in terms of efficacy and safety.

In previous studies, the EMR-C method required a shorter time for the resection of rectal NETs than ESD: 4.2 (2.0) vs. 19.8 (11.3) minutes in one study^13^, and 9.52 (2.14) vs. 24.79 (4.89) minutes in another^24^. While an endoscopic resection time of less than 10 min is acceptable in daily clinical practice, our study suggests that ASEMR is more efficient than EMR-C in terms of resection time. We found that the resection time of the suspected or established NETs was significantly shorter with ASEMR than with EMR-C (3.10 [1.83] vs. 4.57 [2.31] min), which was probably because additional devices and preparation were not required for ASEMR. Although the difference of resection time between EMR-C and ASEMR did not seem to be clinically significant, our data suggest that ASEMR may be convenient and can be selected as a therapeutic option for small rectal NETs. The prices of the snares used for EMR-C and ASEMR are the same at $ 58 (64,240 won), and the usage of caps adds a cost burden to the hospital, not to the patient, in Korea. Therefore, there is no difference in the patient charge between ASEMR and EMR-C in the current study.

In this study, two NETs were not successfully resected using the ASEMR method. One failed ASEMR was performed using a thin and less-stiff snare, which had a horizontal faced position weight lower than that of the other oval stiff snare used in this study (2.96 vs. 16.1 g). To use the leverage action of ASEMR to remove a rectal NET, the anchored snare should be stiff enough to prevent bending while snaring. Of 44 ASEMR attempts using a stiff oval snare, only one ASEMR failed, and this was because the anchored snare-tip was released before completely capturing the rectal NET. Therefore, to ensure en bloc resection of ASEMR, the anchoring of the snare-tip should persist until the snaring is completed. If the snare tip is fixed well at a mucosal slit and the snare does not slip during an ASEMR attempt, we think that ASEMR could be selected as a therapeutic procedure. The two failed ASEMR cases were successfully treated after immediate conversion to ESD. We suggest that immediate conversion to ESD is an effective rescue therapy in the case of failed ASEMR for a rectal NET.

In this study, a 13 or 15 mm snare size was used for ASEMR, and the ASEMR group included just two cases larger than 10 mm in size (11 and 12 mm). Both lesions were completely resected using ASEMR with a 13 mm oval stiff snare, and their deep safety resection margins were 230 and 1900 μm, respectively. Although the pathology-determined size of the NETs and deep safety resection margins were not correlated in the ASEMR and EMR-C methods in our study, additional studies are necessary to establish the lesion-size limitations determining the suitability of ASEMR.

Recently, other techniques for endoscopic resection of rectal NET were introduced. Underwater EMR (UEMR) is a novel modified EMR method that can promote the lifting and floating away of a submucosal tumor from muscularis propria by buoyancy^25^. In a retrospective study analyzing small rectal NETs (≤ 10 mm), UEMR achieved 86.1% of histologic complete resection rate comparable to ESD. The resection time was significantly shorter than ESD (5.8 ± 2.9 vs. 26.6 ± 13.4 min, p < 0.001), and UEMR-related complications were not occurred^26^. Although ASEMR in the present study shows a slightly higher complete resection rate (94.1%) than UEMR in the previous study, a head-to-head comparison based on two different studies is not appropriate. Theoretically, given that most rectal NETs invade the submucosa, the lack of submucosal fat tissue beneath the NETs may restrict the tissue buoyancy for UEMR^27^. However, this theoretical limitation may not affect the completeness of resection in UEMR because UEMR can facilitate en bloc snaring of NETs by avoiding distension of rectal lumen. At this moment, both ASEMR and UEMR seem feasible to remove small rectal NETs. In a recently published German multicenter study (n = 40), clip-assisted endoscopic full thickness resection (EFTR) achieved 95.0% of histologic complete resection with median procedure time of 18.5 min (range, 7–60 min)^28^. Five patients (12.5%) experienced minor adverse events such as periprocedural bleeding (n = 4) and device snare rupture (n = 1). In comparison with transanal microsurgery, EFTR showed higher histologic complete resection rates (100% [n = 14/14] vs 92.3% [n = 12/13]) and shorter mean procedure time (19.2 vs 48.9 min)^29^. We think EFTR can be selected for a rectal NET with suspicious deep submucosal invasion which can be difficult to perform complete resection by EMR or ESD. Moreover, the procedure time is much longer than modified EMR and the price of the EFTR devices is more expensive. Additional studies are needed to investigate the outcomes of two novel procedures as endoscopic treatment of rectal NETs.

In comparison with ASEMR in the present study, EMR-P and EMR-L also achieved similar complete resection rates with minor complication in the previous studies^14,30,31^. EMR-P can also be used for the resection of a large scarred polyp with an acceptable recurrence rate (16%) while avoiding surgery^32^. However, EMR-P may take more procedure time than ASEMR due to making a circumferential incision of the mucosa. EMR-L needs a band ligation device in addition to conventional snare. Meanwhile, ESD showed high complete resection rates (82.6%–100%) and could be indicated as a salvage therapy in patients with residual disease after standard polypectomy^14,33^. However, ESD requires a relatively long learning period and carries a higher risk of complications compared to EMR^12^.

This study has several limitations. First, there may be a selection bias due to the retrospective study design based on the experience of a single endoscopist in a tertiary referral center. In addition, choice between ASEMR and EMR-C may also cause selection bias. The reason for implementing ASEMR from March 2019 is due to the issue of reimbursement for EMR-C in the present study. Because the crescent type snare for EMR-C is not appropriate to perform conventional EMR, we should use an additional snare to resect synchronous colorectal adenomas. Because using two snares in a single session of procedure is not reimbursed by the national health insurance system of Korea, the second snare adds a cost burden to our center. Thus, we performed ASEMR that can remove rectal NETs as well as other synchronous adenomas with one snare. Considering that there was no significant difference in baseline and lesion characteristics except for patients’ age between ASEMR and EMR-C groups, the effect on selection bias may not be significant. Though it is difficult to generalize our findings due to small sample size, given the low incidence of rectal NET and the small patients’ population in the previous published literatures^14^, we believe that our study could provide meaningful clinical implications. Multicenter prospective studies should be performed to establish the therapeutic efficacy and safety of ASMER for rectal NETs in comparison with other modified EMR techniques. Second, the long-term outcomes of endoscopic resection could not be acquired because of the short follow-up periods. However, in a previous study, neither metastasis nor recurrence occurred during a median follow-up period of 67.5 months without additional surgery being performed for those patients with NETs showing a positive resection margin (n = 2) or lymphovascular invasion (n = 42)^34^. Another multicenter study showed an extremely low local recurrence rate (0.74%) and no distant recurrence during a median follow-up of 45 months, although the complete resection rate of rectal NETs was 63.6%^35^. Therefore, the long-term outcomes of rectal NETs treated with ASMER should be as favorable as those of the previous studies. Third, both ASEMR and EMR-C were performed by a single expert endoscopist who had expertise in EMR, EMR-P, EMR-C, and ESD. Though learning curve of ASEMR has not been reported so far, previous published literatures on ASEMR have been analyzed primarily for large colorectal polyps (≥ 10 mm), and small NETs (≤ 10 mm) are expected to be much easier than that of large colorectal polyps^19,20,36^. Furthermore, ASEMR is a minimally modified EMR but is actually very similar to conventional EMR except for making a small mucosal slit and anchoring a snare there, we believe that endoscopists skillful at EMR can easily learn ASEMR.

## Conclusion

ASEMR was less time-consuming than EMR-C, and showed a similar therapeutic efficacy and safety profile for the treatment of small suspected or established rectal NETs. Therefore, we consider ASEMR to be feasible for the removal of small rectal NETs. Additional prospective multicenter studies are warranted to determine the effectiveness, efficiency, and safety of ASEMR for non-metastatic small rectal NETs.

## Supplementary Information

Supplementary Video 1.

Supplementary Video 2.

Supplementary Video Legends.
