# Supplementary material for: Anchoring the snare tip is a feasible endoscopic mucosal resection method for small rectal neuroendocrine tumors
Source: Sci Rep. 2021 Jun 21;11:12918. doi: 10.1038/s41598-021-92462-y (PMC8217176; doi:10.1038/s41598-021-92462-y)
Supplement: Supplementary file 3 — Supplementary Video Legends. [file 41598_2021_92462_MOESM3_ESM.docx]

**Supplementary Materials**

**Video 1.** Anchored Snare-tip Endoscopic Mucosal Resection for Rectal Neuroendocrine Tumors

**Video 2.** Anchored Snare-tip Endoscopic Mucosal Resection for Rectal Neuroendocrine Tumors, Two Failed Cases

**Title**

Anchoring the snare tip is a feasible endoscopic mucosal resection method for small rectal neuroendocrine tumors

**Author lists**

Jeongseok Kim^1,2^, Jisup Kim^3^, Eun Hye Oh^1,4^, Nam Seok Ham^1^, Sung Wook Hwang^1^, Sang Hyoung Park^1^, Byong Duk Ye^1^, Jeong-Sik Byeon^1^, Seung-Jae Myung^1^, Suk-Kyun Yang^1^, Seung-Mo Hong^3^, Dong-Hoon Yang^1^

^1^Department of Gastroenterology, Asan Medical Center, University of Ulsan College of Medicine, Seoul, Korea

^2^Department of Internal Medicine, Keimyung University School of Medicine, Daegu, Korea

^3^Department of Pathology, Asan Medical Center, University of Ulsan College of Medicine, Seoul, Korea

^4^Department of Gastroenterology, Haeundae Paik Hospital, Inje University College of Medicine, Busan, Korea
